# Application of Response Surface Methodologies to Optimize High-Added Value Products Developments: Cosmetic Formulations as an Example

**DOI:** 10.3390/antiox11081552

**Published:** 2022-08-10

**Authors:** Francisco-Javier Leyva-Jiménez, Álvaro Fernández-Ochoa, María de la Luz Cádiz-Gurrea, Jesús Lozano-Sánchez, Rodrigo Oliver-Simancas, M. Elena Alañón, Ines Castangia, Antonio Segura-Carretero, David Arráez-Román

**Affiliations:** 1 Department of Analytical Chemistry and Food Science and Technology, University of Castilla-La Mancha, Ronda de Calatrava 7, 13071 Ciudad Real, Spain; 2 Regional Institute for Applied Scientific Research (IRICA), Area of Food Science, University of Castilla-La Mancha, Avenida Camilo Jose Cela 10, 13071 Ciudad Real, Spain; 3 Department of Analytical Chemistry, Faculty of Sciences, University of Granada, Fuentenueva s/n, 18071 Granada, Spain; 4 Department of Food Science and Nutrition, Faculty of Pharmacy, University of Granada, Campus of Cartuja, 18071 Granada, Spain; 5 Deparment of Scienze della Vita e dell’Ambiente, University of Cagliari, Via Ospedale 72, 09124 Cagliari, Italy

**Keywords:** green technologies, cosmeceuticals, phenolic compounds, experimental design, supercritical fluid extraction, pressurized liquid extraction, microwave-assisted extraction, ultrasound-assisted extraction, enzyme-assisted extraction

## Abstract

In recent years, green and advanced extraction technologies have gained great interest to revalue several food by-products. This by-product revaluation is currently allowing the development of high value-added products, such as functional foods, nutraceuticals, or cosmeceuticals. Among the high valued-added products, cosmeceuticals are innovative cosmetic formulations which have incorporated bioactive natural ingredients providing multiple benefits on skin health. In this context, the extraction techniques are an important step during the elaboration of cosmetic ingredients since they represent the beginning of the formulation process and have a great influence on the quality of the final product. Indeed, these technologies are claimed as efficient methods to retrieve bioactive compounds from natural sources in terms of resource utilization, environmental impact, and costs. This review offers a summary of the most-used green and advanced methodologies to obtain cosmetic ingredients with the maximum performance of these extraction techniques. Response surface methodologies may be applied to enhance the optimization processes, providing a simple way to understand the extraction process as well as to reach the optimum conditions to increase the extraction efficiency. The combination of both assumes an economic improvement to attain high value products that may be applied to develop functional ingredients for cosmetics purposes.

## 1. Introduction

During the last decades, there has been a growing interest by consumers to acquire healthy, safe, sustainable, and functional products which increase their quality of life, having a special mention for those used for self-care products. At the same time, the life population style has promoted an increase in the commercialization and consumption of processed food. This trend has caused an increment in the production of huge amounts of by-products, which still conserve bioactive compounds, derived from the manufacturing processes which, currently, are estimated to be around 1.3 billion tons worldwide [1]. For this reason, the current sustainable policies promoted by the European Union, such as the Circular Economy Action Plan (CEAP) [2], are implementing actions to improve the use of resources by reducing food waste, leading to the development of multiple products with high added value from agri-food by-products.

For example, cosmeceuticals, which are topical products containing bioactive ingredients with pharmacological benefits, are high value-added products that have caught the attention of consumers in recent years. The gain in market share and consumer acceptance is due to the beneficial effects on consumer health but also because they favor the sustainability and revaluation of agri-food by-products [3].

Recent studies have revealed the beneficial effects of different natural compounds from food by-product sources, such as phenolic compounds, on skin health [4,5,6]. That is why the latest trends have been focused on developing new cosmetics functionalized with bioactive compounds with the aim to be used as a therapeutic alternative in the treatment of several topic ailments or to improve skin care [7]. Generally, there are difficulties for the extraction of the compounds with a high yield that allow having the compounds in an adequate concentration to exert their bioactive properties. For this reason, the development of new extraction technologies is playing a fundamental role, as they allow the achievement of adequate extraction yields of the compounds of interest [8].

To achieve this purpose, different technologies have been developed to improve the extractive capabilities and to reduce the environmental impact of conventional techniques [9,10]. In this sense, pressurized liquid extraction, supercritical fluid extraction, microwave-assisted extraction, enzyme-assisted extraction, or ultrasound-assisted extraction are the most used green extraction technologies. These advanced extraction techniques have shown greater extraction efficiency compared to conventional ones (maceration, infusion, decoction, or Soxhlet extraction) [11,12,13]. In recent years, the advanced extraction methodologies have gained importance in the cosmeceutical field, since they allow the conducting of extraction procedures expending lesser times, low solvent consumptions, and also lesser energy spent offering relative selectivity in the extraction of compounds and higher yields, thus becoming relevant environmentally friendly methodologies to obtain high quality extracts [14]. With the purpose of exploiting the full potential of these techniques and performing the extraction procedures in an efficient way, recent research has focused on optimizing the recovery of bioactive compounds from by-products. In this way, the design of experiments is a great statistical tool to achieve efficient extraction processes. In addition, it presents advantages to optimize the formulation processes of new cosmetics as well as the incorporation of new ingredients from agri-food by-products with a reduced number of tests, achieving the objective of favoring sustainability and reducing the costs and time of developing cosmeceutical products [11,15,16,17].

In this review, the concepts and applications of the design of experiments (DoE) have been compiled to know how different mathematical models may be applied to choose the best experimental conditions to optimize any extraction procedure with the purpose of providing high quality extracts to be incorporated into cosmeceutical products. Moreover, the current trend related to the incorporation and evaluation of agri-food by-product extracts into cosmetical matrixes are summarized in this work.

## 2. Advanced Extraction Methods to Develop Functional Ingredients

In pursuit of natural sources that provide beneficial effects that may be used as ingredient in cosmetics, industries have focused their efforts on several botanical species or agri-food by-products, since these natural sources have been reported to provide several properties that are beneficial for skin health [18,19]. Traditionally, the extraction and isolation methods to retrieve these beneficial compounds were performed by means of solid–liquid extraction techniques. However, this methodology presents many drawbacks related to energy, time, and solvent consumptions. For these reasons, some new technologies have been developed in order to improve the recovery of beneficial compounds from natural sources. Furthermore, these technologies allow the use of generally recognized as safe (GRAS) solvents that are also authorized for cosmeceutical development, such as ethanol or water [20,21]. Moreover, these advanced technologies provide a better resource utilization since they provide greater amounts of extract using less samples.

However, the obtainment of functional ingredients from natural sources may be a complex task due to the broad variety of compounds in their composition. In this sense, the application of DoE is interesting to provide detailed information about the effects of different factors during the extraction procedure and to help in achieving the greatest recovery of bioactive compounds. Therefore, the combination of these novel technologies and DoE may provide a high enhancement in the bioactive compounds’ retrieval, inducing a reduction of the cost production and a better resource utilization and, consequently, a great advance in the functional ingredients’ production.

These innovative strategies are characterized by the possibility of using GRAS solvents and the application of energy shaped like pressure or heat, causing physicochemical changes in solvents that improve the release of phytochemicals from cells towards extraction solvents [22]. In this sense, these novel technologies differ from each other in the way that they transmit this energy. Then, in this section, a brief summary of technology principles and the main variables which affect the recovering of bioactives are exposed.

**Figure 1:**
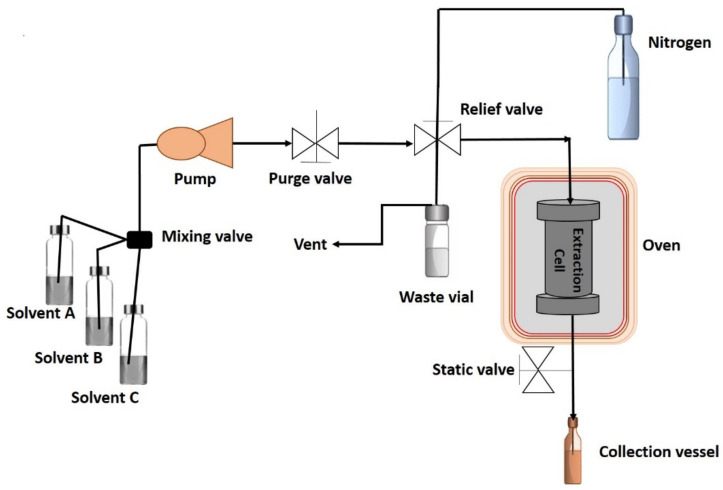
Diagram of pressurized liquid extractor.

Pressurized liquid extraction (PLE) is a relatively faster extraction system that combines two principles: (a) increased temperature and pressure, and (b) interaction between solvent and matrix compounds. This extraction method has been acquiring popularity since it is more efficient in terms of time, solvent usage, and recovery of compounds than traditional extraction techniques [23], which reveal it to be a safe and fast technique. Pressurized liquid extraction uses temperatures comprised between room temperature up to 200 °C and a pressure around 110 bars. These high temperatures applied at a high-pressure atmosphere achieve the increasing of the extracting solvent power by enhancing the diffusivity, solubility, and mass transfer rates. These facts enable the breakage of different bounding forces, such as dipole-dipole or H_2_-bounding, improving the transfer of target compounds from the source to the solvents. During an extraction process, the sample is introduced into an extraction cell, generally of stainless steel. Then, as shown in Figure 1, the extraction cell is introduced in an oven and the solvent is pumped by a pump and pressurized into the extraction cell. In this sense, the heat transfer inside is achieved by radiation, and consequently the center of the cell needs more time to reach the set temperature than the external parts, being the most disadvantage of this technology since the thermosensitive compounds are recovered in less amounts or may suffer thermal degradations, which reduce the bioactivity of some phytochemicals [24]. Nevertheless, PLE accomplishes a relative selective extraction of the compounds in a rapid and easy way. The most influential factors which have effects in PLE technique are temperature, pressure, solvent composition, and extraction time [25].

**Figure 2:**
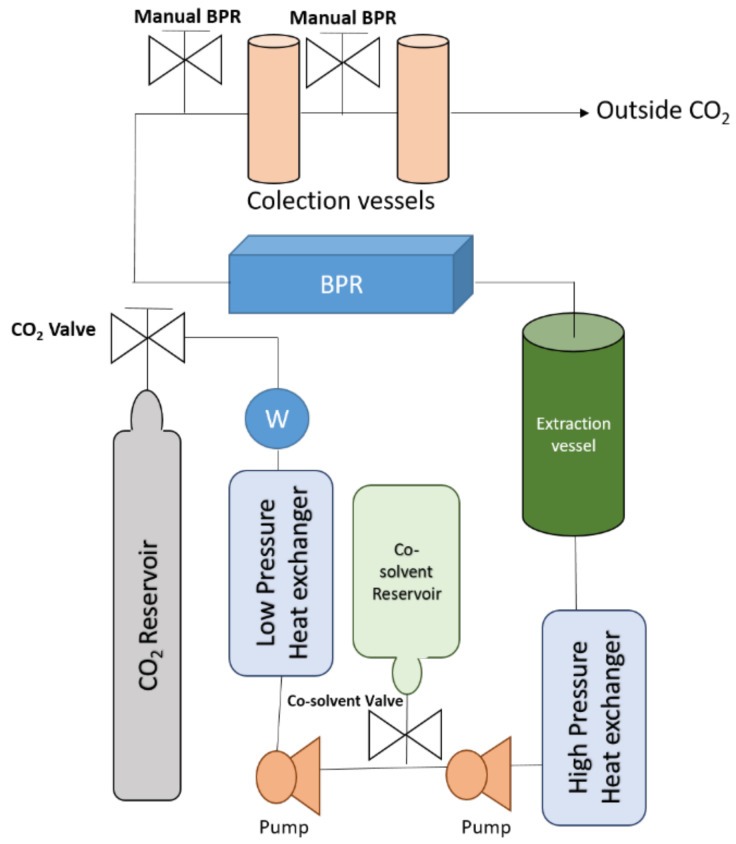
Diagram of supercritical fluid extractor. W: chiller; BPR: back pressure regulator.

Supercritical fluid extraction (SFE) is claimed as a selective extraction method which uses cheap solvents such as ethanol or CO_2_. Supercritical fluids are fluids which are subjected to pressure and temperature conditions above their critical points. This situation gives different properties to the solvents in terms of diffusivity and viscosity, making it that supercritical fluids have features of both liquids and gases [26], enhancing the solvent penetration in the sample and achieving a better extraction of target compounds. Overall, the most used solvent is CO_2_, since it is a cheap solvent with low critical points (73.8 atm and 31.1 °C) and it is easy to remove from the extract due to at atmospheric condition as gaseous. However, the apolar character of this solvent makes mandatory the use of a modifier to obtain polar compounds such as phenolic compounds. A modifier is a solvent which adjusts the polarity of the mix solvent and improves the recovery of the polar compound. Ethanol is the most used modifier in phenolic compounds extraction during SFE extractions [27,28]. Figure 2 displays a brief explanation of a supercritical fluid extractor. In spite of the fact that supercritical fluid extraction allows a selective extraction of compounds from natural sources using cheap solvents, the time spent during the extraction procedure is very long. The outcomes of this technology depend directly on the pressure, temperature, and modifier used [25].

**Figure 3:**
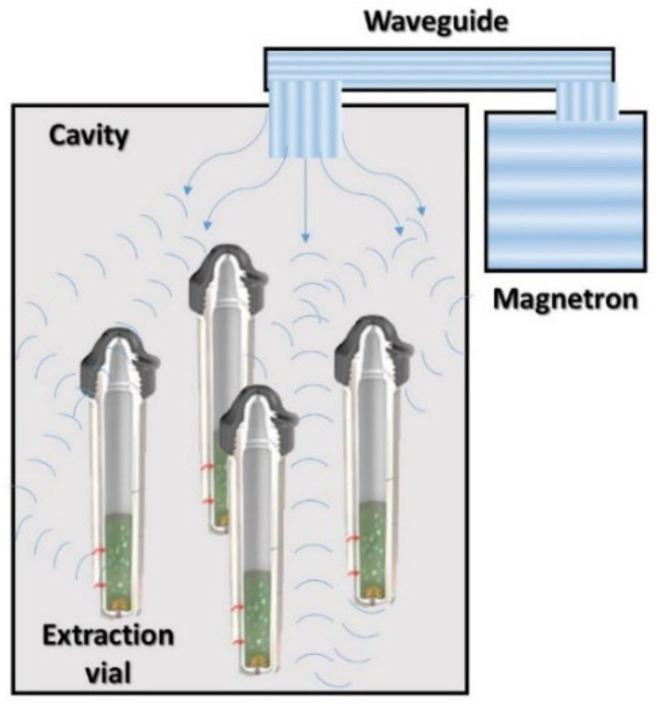
Diagram of a microwave-assisted extractor.

Microwave-assisted extraction (MAE) is an innovative technique to obtain the enriched extract from natural sources, which is characterized by being a simple, cost-effective, and rapid technique (Figure 3). Microwave-assisted extraction is usually used to recover thermosensitive compounds from plants since its heating principle consists of ionic conduction and dipole rotations of molecules caused by two oscillating perpendicular fields, electric and magnetic, which generate frictions and collisions between molecules inducing heat. In plant matrices, this heat achieves the evaporation of the water in plant cells causing a swelling and breakage of cells, promoting the releasing of target compounds into the solvents. The waves applied are ranged from 0.3 to 300 GHz [24,29]. Moreover, this technology has been used to attain high quality extracts with similar or better yields compared to conventional extraction methods spending less solvents, time, and samples [26]. The most important advantage is the ability to recover thermolabile compounds in short periods of time. Furthermore, it is an easy-to-use technique which provided a relative selective extraction of target compounds. The most influential parameters to consider during a microwave-assisted extraction experiment are extraction time, solvent composition, temperature applied, and ratio sample-solvent.

Ultrasound-assisted extraction (UAE) has been revealed as a cheap green extraction method which may be considered as a versatile, simple, safe, rapid, and highly cost-effective technique used to attain bioactive compounds such as phenolic compounds located in the vacuoles of the plant cells [30]. An ultrasound is a type of sound wave between 20 kHz and 100 MHz, beyond human hearing. Like other waves, it passes through a medium making a cavitation phenomenon, which consists of a successive process of compression and expansion of the medium, causing the production, growth, and collapse of the created bubbles that may reach up to 4720 °C and 1000 atm [9]. As a consequence of this, bubbles generate a micro-jet directed to the surface and abrasion of the surface, breaking the cell walls, and allowing the release of phytochemicals into the solvent [30]. This technique could be used for extraction either with an ultrasonic bath or ultrasonic probe. The main differences between both systems lie in the amplitude supported (20 kHz for probe and 40 kHz for baths), the number of samples treated at the same time (ultrasonic bath allows a greater number of samples), the cavitation efficiency (higher using ultrasound probe, as a higher intensity is introduced to a specific area), and the duration of the extraction process (higher in bath, as the intensity is provided in a wider area) [31]. Moreover, an ultrasound probe may produce metallic residues in the sample due to its degradation, making good maintenance necessary to reduce this inconvenience. The advantages of this extraction method include decreasing extraction time, energy, and solvent consumption. Because of its characteristics, this extraction method causes an effective mixing between solvent and sample, reduced extraction temperature, selective extraction, and increased recovery of the bioactive compound coupled with an ease of utilization [32]. However, during UAE, reactive oxygen species (ROS) are generated by sonolysis, which may result in the degradation of some interesting compounds. This event occurs when the water molecules are broken due to the energy supplied by the ultrasound generating H· and OH· [33]. These radicals are neutralized by phenolic compounds found in the extraction solvents [34]. During experimental conditions, the most influential factors are temperature, pressure, frequency, and time of sonication.

Another method that applies hydrodynamic cavitation is negative pressure cavitation-assisted extraction (NPC). This technique creates an intense cavitation phenomenon via a negative pressure created by vacuum pump and a continuous airflow, maintaining turbulence in the extraction vessel that corrodes the surface of solid particles. This situation promotes the turbulence, collision, and mass transfer between the extraction solvent and solid matrix when air is continuously added into the system via the vale, and facilitates the migration of compounds from the sample to extraction solvents [35,36]. This technique allows working at moderate temperatures and with an oxygen-free atmosphere. In addition, this technique may be used in combination with other advanced techniques (MAE or EAE [37]). For these reasons, it is suitable for recovering thermosensitive compounds and easily oxidized compounds as well. Moreover, it is a time saving, economic, energy efficient, and eco-friendly methodology for extracting bioactive compounds from plants. Nevertheless, its recent implementation has only been carried out on a lab scale, and therefore it is necessary to test it on an industrial scale [35]. The main factors that have a relevant influence on the NPC are temperature, type and proportion of solvents, pressure intensity, extraction time, liquid-to-solid ratio, and presence of dissolved gas.

Some phytochemicals from plant sources are bounded by hydrogen or hydrophobic links and kept in cell walls, hindering their release into the solvent. In addition, the recovered phytochemicals may be bounded to other components that make their isolation difficult. In this sense, enzyme-assisted extraction (EAE) has been revealed as a novel and effective technique to bring phytochemicals out and improve their retrieval [38]. Enzymes such as pectinase, xylanases, proteases, or amylases are examples used in this technique to extract bioactive compounds from agri-food by-products [39]. These enzymes induce hydrolysis on structural polysaccharides and other components in the cell walls. Therefore, the enzyme composition and concentration are important factors to consider during an enzyme-assisted extraction procedure. Moreover, the particle size of the sample, solvents used, the pH and temperature of dissolutions, the time of procedure, and the solid–solvent ratio also have a determinant effect during the extraction [40,41]. EAE can also be combined with other advanced extraction techniques, improving the efficiency of extraction processes and the quality of extracts [42].

Additionally, electric field-assisted extraction (PEF) has initially been applied for food preservation since it achieves enzymatic and antimicrobial inactivation. Currently, it is applied to recover valuable compounds from several natural sources [43,44]. This non-thermal technology is based on the electroporation phenomenon on cell membranes that occurs when they are exposed to a moderate or high electric field (0.1–50 kV/cm) and relatively low energy (1–20 kJ/kg) during short pulses (µs). This phenomenon leads to a loss of influx and efflux transport selectivity, increasing the permeability and promoting the penetration of a solvent into the cell, and, consequently, causing the diffusion of solubilized valuable compounds from the cells [45]. A variation of this technique is also used for the recovery of bioactive compounds, and is called high voltage electrical discharge (HVED) [46]. The PEF technique is characterized by its low energy consumption, continuous operability, and short extraction times, increasing the sustainability of the technique. The main advantages of this extraction technique lie in the low degradation of thermosensitive compounds and the ease of extract purification [47]. The efficiency of the process strictly depends on the field strength, specific energy input, pulse number, pulse duration, and treatment temperature [24].

## 3. DoE: From Concept to Product Development

In the recovery of bioactive compounds, it is common to perform many tests with the purpose of improving the manufacturing of functional ingredients, for example, to attain the best yield during an extraction procedure. The outcomes of these assays are usually analyzed by a trial-and-error basis requiring a large number of tests that have an important impact on the economic cost of the development of products. To address these limitations, the use of DoE has become an efficient alternative because it allows a careful planning of the experiments in advance with the purpose of knowing the optimal conditions of a process using a reduced number of tests.

Therefore, this methodology consists of establishing which experiments should be ideally performed with the purpose of knowing what variables affect a certain process, as well as obtaining the optimal working conditions after the appropriate statistical tests [48]. Therefore, the design of experiments is a set of tests carried out to obtain knowledge about a system or process, producing substantial improvements that facilitate the achievement of the desired objective.

### 3.1. Basic Principles of DoE

With the purpose of understanding the advantages provided by the design of experiments and evaluating their results, the knowledge about the different types of designs as well as the statistical parameters used for their evaluation is necessary. In this scenario, an experimental design has three elements that are mandatory: responses, factors, and levels. The response variable, or dependent variable, is the result measured after applying the different experimental conditions. All the experimental conditions should be addressed to obtain the best value for the selected response variable (maximizing, minimizing, or to achieve a specific value of the response). Factors or independent variables are the variables set by researchers during the experiments for the evaluation of their effects on the response variable. The number of factors depends on the procedure, equipment, and principles in which the selected technique is based to generate the functional ingredient. In addition, factors are defined by levels, or values, assigned to each independent variable which are represented in a range between −1 (minimum value) and 1 (maximum value), with 0 being the middle value. Combining different factors under a specific type of experimental design, a variable number of experiments will be procured for evaluating the potential of the selected technique. Thus, each experimental point comprises a blend of factor levels [49].

It is necessary to remark that to keep the effectiveness of this methodology is important to delimit the responses, factors, and levels to monitor, because the total number of experiments depends directly on the number of selected levels and factors [50]. Therefore, the selection of the factors and their levels requires an exhaustive prior study by the researchers on the conditions and parameters that can influence the process to be optimized.

Considering the broad combination of factors, levels, and response variables to evaluate, the DoE can be classified according to the objective of the experiment. Industries are focused on designs which enable the evaluation of the effects of factors on response variables, and to optimize a process applying the smallest possible number of experiments.

### 3.2. Types of DoE

The response surface methodology involves the three following aspects: design, model, and optimization. The first term is related to a mathematical model that allows summarizing the behavior of the response variables under the evaluated experimental conditions. This mathematical model may establish the linear interactions and quadratic interactions of independent variables following first or second order equations (Equations (1) and (2), respectively):

(1) $$Y={\;\beta }_{0}+\overset{k}{\underset{i=1}{\sum }}\beta_{i}x_{i}$$

(2) $$Y={\;\beta }_{0}+\overset{k}{\underset{i=1}{\sum }}\beta_{i}x_{i}+\overset{k}{\underset{i=1}{\sum }}\beta_{\mathrm{ii}}x_{i}^{2}+\overset{k}{\underset{i=1<}{\sum }}\overset{k}{\underset{j=1}{\sum }}\beta_{\mathrm{ij}}x_{i}x_{j}\;$$

Y represents the response; β_0_ is a constant coefficient that fixes the response at the central point of the experiments, and β_i_, β_ii_, and β_ij_ are the regression coefficients of the linear, quadratic, and interaction terms, respectively; x_i_ and x_j_ represent the value of independent variables [13]. In this sense, the first order design is used when only the principal effects of factors are evaluated, whereas the second order design involves an individual study of factors but also the interaction between them and their quadratic effects.

The model points out the level adjustment of the mathematical regression. For it, different parameters are used such as model fitting, lack-of-fit tests, residues, and predicted and determination coefficients to verify the fitting of the proposed model. These parameters are explained in more detailed in the next section.

Once the model fitting is verified, the optimization enables the maximization of the evaluated response. For that purpose, the results obtained after performing each point of the experimental design are plotted in a graph, whose surface describes the dependent variable behavior, allowing the discernment of the combination of levels that results in an optimal response value [51].

The simplest experimental designs are denominated factorial designs (2^k^ and 3^k^). These designs consist of k factors fixed at two or three levels, respectively. They are the basis of more complex designs and usually used as a first attempt to discern where the optimal experimental range and the factor set may be. These designs may be useful to identify the most influential factor during a process, facilitating its selection for later use as factors in the optimization process. For instance, the Plackett–Burman design provides a fast and effective way to identify the relevant factors among a large number of variables. This design may be used as a first step in the optimization processes since it provides information about important factors during a process with a reduced number of experiments [52]. In this sense, the application of a combination of PBD and optimization designs may represent an intelligent way to achieve the stated objectives and obtain greater efficiencies from the technique used, spending less time and resources [53].

Between the two main types of mathematical models (Equations (1) and (2)), the second order models are the most common in extraction procedures for the development of functional ingredients from agri-food by-products since they are more useful to optimize complex processes. Moreover, this kind of model gives more detailed information about the effects of factors, their interaction, and their quadratic effects [54,55]. The main condition of these designs is that the factors should be considered in at least three levels (−1, 0, 1) to estimate the curve generated on the response surface. In this sense, the most used experimental design based on response surface methodologies (RSM) are the Box–Behnken design (BBD) and Central Composite design (CCD).

**Figure 4:**
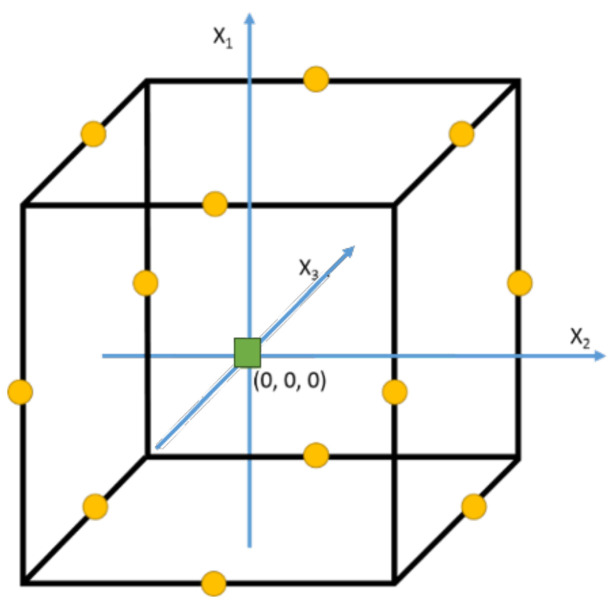
Graphical representation of three factor BBD. Circles are experimental points and square is the central point. X_1_, X_2_, and X_3_ are the factors evaluated.

On the one hand, the BBD is used when three or more factors are considered. This kind of design does not include experimental points on the vertexes, and consequently, all factors cannot be simultaneously established at their highest or lowest values (−1, −1, −1) or (1, 1, 1), as shown in Figure 4. Moreover, at least one of the factors is fixed at the middle range during each run, as shown in Table 1 [56].

This situation’s results are useful when extreme conditions may not be performed, for example, when the extraction solvent is evaporated at a high temperature. Conversely, it is a rotatable or nearly rotatable design since it does not include these extreme conditions. Therefore, the prediction of the behavior of the evaluated responses will be invariant if the central conditions are kept, although the values of the levels are changed.

**Figure 5:**
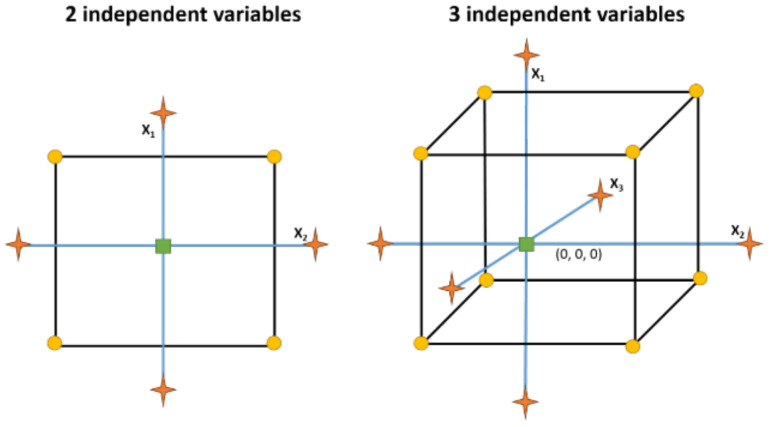
Graphical representation of two and three factor CCD. Circles are experimental points and square is the central point. Stars are axial points. X_1_, X_2_, and X_3_ are the factors evaluated.

On the other hand, the CCD is broadly used due to its high flexibility. In fact, the experimental results early attained in a factorial design may be used in a CCD performing only the axial points, as displayed in Figure 5, thus minimizing the waste of resources. Moreover, this design, as with the BBD, contains at least two replicates of central points (0, 0, 0) that allow it to know the reproducibility of the experiments. However, the most characteristic parameters are the axial points (−α, α). These points are beyond the minimum and maximum limits of the factors, guaranteeing the curvature of the response surface, and, hence, enabling the establishment of the optimal conditions (Table 2 and Table 3).

In contrast to the BBD, the characteristics of orthogonal and rotatable can be attributed to a CCD finding the difference in the estimation of axial points [57]. It is necessary to remark that an orthogonal design enables the evaluation of the principal, interaction, and quadratic effects in an independent way, making the interpretation of results easier [58].

### 3.3. Data Processing and Statistical Analyses

The data obtained after performing the experimental runs must be statistically analyzed to discern the fit of the model and, consequently, provide an objective prediction of the response behavior, the independent variable effects, and a reliable optimization of the process. In this sense, several parameters are used to analyze the model. The first parameter to assess the fitting quality of the model is the determination coefficient (*R*^2^). The determination coefficient reveals the proportional variability of data that can be explained by the mathematical model. The values of this parameter are comprised between 0 and 1, revealing a good fitting when determination coefficient values are over 0.8 [13]. Nevertheless, when there are many terms in the model, it is preferable to use the adjust determination coefficient ($R_{adj}^{2}$), being lower than *R*^2^ since $R_{adj}^{2}\;$ penalizes the inclusion of terms that do not contribute to explaining the variability. A good fitting is considered when values of $R_{adj}^{2}$ are over 0.7 [59]. Together with these coefficients, the residual plots give a visual representation of the data variation according to the model applied. Another measure to evaluate the model fitting is the lack-of-fit test, which verifies the fitting quality of the model applied [60]. In this sense, a model is fitted when the result of this test is not significant (*p*-value higher than 0.05) [61]. In first order models, *p*-values of the lack-of-fit test less than or equal to 0.05 reveal the presence of a curvature in the model, and, consequently, a lack-of-fit towards the proposed model [48]. Moreover, the model adequacy is also used as an approach to discern the good design choice, revealing a good approach when the *p*-value is less than or equal to 0.05 [62].

Once these parameters have been evaluated revealing a good fit, the proposed model can be used to predict the behavior of the response variable if the conditions are within the experimental range. Furthermore, an objective and reliable optimization may be performed by searching in the surface outlined by the model. This optimization can be focused on maximizing, minimizing, or achieving a target value of a single response variable, or to achieve the optimization of several responses (multiple response optimization). In the case of multiple response optimization, it is necessary to consider that the optimum conditions of both responses may not be the same as the optimum conditions individually. For this reason, it is necessary to fix a simultaneous optimal condition that provides the best results for both responses. To solve this problem, Derringer and Suich suggested the desirability function, which enables the identification of the simultaneous optimum conditions. This function is based on the estimation of global desirability of the responses (the best condition for both responses) in each run. In this sense, it is only necessary to maximize or minimize the global desirability to find the optimal point. The desirability function takes values from 0 to 1, where values next to 1 reveal the best conditions to achieve the proposed optimization [63]. Furthermore, with the purpose of evaluating the factor effects on the response variable’s behavior, an analysis of variance (ANOVA) is performed. To this end, the individual, interaction, and quadratic effects (for a second order model) are individually assessed. In this way, one factor exerts a relevant effect when the *p*-value ≤ 0.05, pointing out a significant impact on the response variable outcome.

### 3.4. Application of DoE to Optimize Phytochemicals Retrieval by Advanced Extraction Techniques from Natural Sources

As discussed above, it is necessary to know the principle of each extraction technique since the application of one extraction method or another depends directly on the characteristic of the phytochemicals that will be recovered. In other words, the huge variety of phytochemicals contained in agri-food by-products, and consequently, their different structures and locations inside the matrix will play a major role in the extraction method selected. For instance, MAE applies the heat promptly from inside the plant cell, decreasing the extraction time, and hence, reducing the degradation of thermolabile compounds as anthocyanins [64]. On the other hand, the application of PLE enables the extraction of more complex polyphenols, such as tannins or lignans, but also lowers thermosensitive phenols [65]. Additionally, SFE is generally used to attain essential oil fractions or to obtain thermosensitive non-polar compounds, such as terpenes or carotenoids [66,67], but also is used to recover some thermolabile phenolic compounds [26]. Considering these relationships between phenolic compounds’ retrieval and extraction techniques, it is important to choose the most influential factors during the extraction procedures, as well as to delimit the factor level to achieve the desired outcomes. In this sense, Table 4 compiles a summary of recent works where DoE is based on RSM, and advanced extraction technologies are both applied to obtain functional ingredients. Thus, the exposed works are mainly focused on the attainment of enriched extracts in bioactive compounds, such as phenolic compounds or carotenoids, with antioxidant or enzymatic inhibition capacities, which may have a potential use in cosmetics.

According to Table 4, the most influent factors in a MAE process are the extraction time, the solvent:sample ratio, the solvent composition, and the power applied. The last one is directly related to the temperature of the process [97,98,99]. Concerning UAE, the most relevant factor is the ultrasonic intensity [71]. Nevertheless, the time spent during the sonication, the solvent:sample ratio, and the solvent composition are also influential factors [73,93]. NPC is influenced by the solvent:sample ratio, negative pressure, and ethanol concentration [35,86]. However, the extraction efficiency may be improved when it is used coupled to other technologies such as the microwave, enzyme, or ultrasound, being that it is necessary to adjust the conditions of both methodologies to obtain targeted compounds [35,37,86]. Due to the fact that PLE is performed in equipment that usually sets the working pressure at 100–110 bars [11,75], temperature and solvent composition are the factors that exert a major effect on the evaluated responses [76]. However, the number of extraction cycles or the static extraction time are also usually evaluated [74,100]. As previously mentioned, the most common solvent used in an SFE is CO_2_, which provides large recoveries of non-polar compounds. Therefore, the flow and the type of modifier are also important in this technique in order to recover more polar compounds [92,101]. For these reasons, the most common factor assessed during SFE processes are the carbon dioxide flow, the temperature, and the work pressure [91,102]. Finally, an acidic or basic extraction medium can considerably affect the efficiency of an enzymatic reaction. In this sense, the correct pH adjustment during an EAE can be decisive to achieve the desired results, as well as the work temperature and the substrate and enzyme concentration [94,95]. Finally, PEF efficiency is mostly influenced by the pulse field strength and number, as well as the ethanol concentration [44,96]. These parameters should be controlled to avoid an increase in the extraction temperature that degrades thermosensitive compounds, and to achieve an efficient breakdown of the cell walls and thus the release of valuable compounds.

Table 4 shows the importance of applying the design of experiments in order to achieve the maximum potential of advanced extraction methodologies that generally are used to optimize the total phenolic content present in extracts obtained from botanical sources [72,84]. Apart from this, the bioactivity of the attained extracts is also checked, being that the antioxidant capacity and total phenolic content are the most considered response variables [80,89,90]. It is necessary to remark that spectrophotometric assays are useful to estimate the antioxidant capacity; however, these are not appropriate for estimating the number of individual compounds or a certain family of compounds, since there may be compounds from different chemical classes that can react in the proposed reaction mechanisms. For instance, the Folin–Ciocalteu reagent, used for total phenolic quantitation, is not specific for phenolic compounds as it can also be reduced by many non-phenolic compounds. Although, this assay is performed in basic conditions (promoting phenolate anion formation), in order to enhance the reduction of the Folin–Ciocalteu reagent by phenolic compounds, and the final quantitation should be corroborated by most selective platforms [103]. In the same line, antioxidant capacity assays (DPPH, TEAC…) should not be used for quantifying specific antioxidant compounds in a sample, since there are a wide variety of compounds (phenolic compounds, carotenoids, vitamins, etc.) that can react with the radicals [103]. However, these non-selective evaluation techniques could be applied to compare the antioxidant potential of whole extracts obtained by different conditions. For these reasons, the use of analytical platforms can enhance the optimization of the individual compound as well as specific compound classes [26]. In this sense, advanced analytical platforms, such as high performance liquid chromatography (HPLC) or HPLC coupled to mass spectrometry, should be used to determine individual compounds, verifying the results obtained by RSM designs since optimal conditions may differ from those proposed when spectrophotometric assays are used [104,105,106]. Moreover, the use of analytical platforms also allows a relatively selective extraction of specific compounds or phytochemical groups, focusing on those bioactive compounds that can offer a high value for cosmetic products [107].

As can be seen in Table 4, not all the studies collected used all the parameters to verify the fit of the mathematical model (*R*^2^, $R_{adj}^{2}$, model adequacy, and lack-of-fit), calling into question its predictive capacity. Hence, the optimization capability of the proposed systems is under discussion. These results may be associated to a non-linear behavior of some evaluated responses. However, if not all the variables studied have a linear behavior, the results obtained are not reliable and therefore cannot be used to optimize the process [108]. For this reason, in the last years, artificial neural networks (ANN) have gained attention to model and optimize processes. This methodology is based on computational and mathematical methods which attempt to simulate the neurological processing ability of the human brain. ANN allow the evaluation of relationships between the factors and the responses’ variables of processes, applying a limited number of experimental measurements. ANN could predict results based on previous data owing to their capacity to learn from observations and create conclusions via the generalization and modelling of complex non-linear behaviors [109]. Nevertheless, this methodology requires a significant computational burden to develop and implement neural networks to achieve optimal performance [110]. Additionally, the complexity of choosing the neural network architecture is high since the network should be adapted for the new response data. Furthermore, many existing systems of computational intelligence are unable to determine the evolving rules by which the systems are developed and can also present the results of their work in natural language terms. Although ANN produce a probing solution, they do not give a clue as to why and how, reducing the reliability in the network [111]. On this scenario, RSM and ANN may be applied in combination to achieve a credible and reproducible optimization of the process, since they would allow the evaluation of both responses, i.e., those with linear behavior and those without. In fact, some works have already optimized several extraction procedures by applying RSM and ANN methodologies, showing that both optimization methods provide similar results and allow a complementation in the results favoring a more complete, credible, and reliable understanding of the behavior [83,112,113].

According to the exposed outcomes, the application of experimental designs (RSM and ANN) combining with innovative extraction techniques to obtain enriched extracts in specific compounds at the lab scale, may improve the scale-up of these procedures in food industries, as well as the knowledge about the effect of several factors leading to a reduction in the economic costs of the industrial processes.

## 4. RSM for Improving the Cosmeceutical Formulations

The growing production of ready-to-consume food causes an excessive increase in the production of by-products, such as leaves, skins, or seeds, which contain bioactive components that can be used for several purposes. As mentioned above, RSM applied to advanced extraction methodologies can help to achieve high quality extracts for use as functional ingredients for different cosmetic purposes. Furthermore, these optimization processes can also be applied to improve the formulation of cosmetic products. Table 5 shows several examples of this type of study. For instance, Poomanee et al. have developed an RSM based on the CCD to optimize the formulation of nanoemulsions containing mango kernel extract for improved acne treatment. In this work, the HLB value and the concentration of PEG-7 glyceryl cocoate had a relevant effect on the droplet size and PDI, decreasing these values when HLB and emulsifier concentrations reached higher values, whereas the Z-potential achieved negative values at these conditions. Moreover, the optimized nanoemulsions presented a great skin permeability, reaching the epidermis and epidermis layer and also passing through them, thanks to the small size that was achieved (20–30 nm), thereby enhancing the delivery of the bioactive compounds from mango kernels in the target zones [112]. On the other hand, kojic acid has been tested as a whitening and anti-melanogenic agent, revealing great results [113]. For this reason, this compound has been incorporated into nanoemulsions, as kojic acid monooleate, which presented better depigmenting effects, in order to improve the penetration and bioavailability along the skin. Therefore, with the purpose of developing stable and little particles’ emulsions, a CCD has been performed to develop high quality nanoemulsions. This optimized nanoemulsion had little particles (104 nm) but they were not homogeneous (PDI between 0.3 and 0.45) which presented less cytotoxicity (IC_50_ > 500µg/mL) and a tyrosinase inhibition of 67% at 20 mg/mL [114]. Additionally, Hübner et al. have succeeded in developing nanoemulsions enriched with cabernet sauvignon grape pomace extract to be applied as skin photoprotectors. In this work, they performed a CCD (11 runs) to develop cosmetic preparations based on emulsions and optimization of the production variables. Despite performing the CCD, they found one of the runs proposed by the CCD as the optimal condition since they used this statistical model to discern the behavior of the experimental factors on each response. In this sense, the formulation developed using 10% of the grape extract and 11.5% of the sunscreen filters revealed the best formulation in terms of the sun protection factor (SPF 16), critical wavelength (λ_crit_ > 375 nm), and UVB transmittance (5 to 25%). This formulation was tested compared with the same formulation without the extract, revealing that it provided up to 18% more protection against UVA radiation than the formulation without the extract. Moreover, the formulation did not induce any adverse reactions of irritability, sensitization, phototoxicity, or photosensitization, thus becoming an interesting ingredient for developing photoprotective cosmeceuticals [115]. Additionally, *Chromoleana odorata* extract has been also incorporated into virgin coconut oil emulsions when applying a CCD for improving the efficiency of the formulation process. The optimal conditions were achieved at 5:95 coconut oil/water ratio, emulsifier concentration 5%, and at a homogenization speed of 7500 rpm. Nevertheless, no bioactivity test was performed with the optimal formulation, and consequently, it cannot determine the skin beneficial effect after application [116].

DoE can not only be used to optimize the process of incorporating extracts, because many times the components of the own formulations are bioactive per se. For instance, a nanoemulsion was developed based on red palm oil which is an important source of vitamin E, having a beneficial effect on skin inflammation [117]. In this work, a CCD was carried out to optimize the smallest and the most homogeneous nanodroplets evaluating the effects of surfactant concentrations (Tween 80 and Span 80), glycerol concentration, and homogenization pressure. Homogeneous little particles (119 nm and PDI 0.28) were obtained after applying 6 wt% of the mixed surfactant (Tween 80/Span 80 (63:37, wt)), 20 wt% glycerol, and 500 bar during homogenization. Unfortunately, this nanoemulsion was not tested by in vitro or in vivo assays to assess the bioactivity of this optimized formula [118]. Moreover, experimental BBDs have been also applied to optimize cosmeceuticals formulations. Yoo et al. performed an optimization procedure based on a BBD to achieve an optimized emulsion based on coconut oil and the incorporation of *Flos sophorae* extract. The active components of this source have shown remarkable anti-cancer, antithrombotic, analgesic, antibacterial, antiviral, anti-aging, anti-inflammatory, anti-allergic, and antioxidant cosmetical properties [119]. In this study, there were evaluated factors, such as emulsification time, emulsification speed, or concentration of emulsifier with the purpose of achieving a stable and homogeneous formulation. Moreover, the viscosity was also considered as a response variable. However, after applying optimal formulation conditions, the bioactive properties of the developed cosmeceuticals were not evaluated.

Based on the current literature, the number of studies addressing the optimization of the formulation process of cosmetic developments is still scarce. In fact, there are only a few studies that address both formulation optimization, the incorporation of bioactive extracts, and their subsequent bioactivity evaluation by in vitro and/or in vivo models. Thus, the potential of these optimization methodologies can be further exploited to develop new cosmeceuticals with the aim of developing more efficient processes and reducing the production costs of these types of products. Unlike extraction processes, for which RSM methodologies have been widely used to optimize compound recovery, there is a lack of information on the application of these methodologies and the incorporation of by-product extracts into cosmetic products, as well as the evaluation of their bioactivity in different models, which should be addressed in the coming years.

## 5. Conclusions

Cosmeceuticals are novel products that have quickly aroused the interest of consumers for their skin health properties. Moreover, their sustainable production from agri-food by-products has achieved an interesting market share in the past few years, postulating them as a relevant self-care product. In this area, advanced extraction techniques have provided concentrated and enriched extracts from by-products to be used in the development of cosmeceuticals. The use of DoE to improve the performance of an extraction method has become an important tool to optimize the best conditions to achieve the desired objectives. CCD and BBD have been demonstrated to be versatile options to enhance the bioactive compounds’ retrieval. In the context of cosmeceuticals, a combination of green technologies and RSM has begun to be applied, in order to optimize the obtaining of functional ingredients from food by-products and to understand the effects of various factors during the extraction process. Although this combination can offer a considerable number of advantages, such as the reduction of production costs, treatments of wastes, or environmental impact, it is necessary to use appropriate statistical tests that validate the fit of the models, since not all the experimental designs applied have used them to justify the correct adjustment of the experimental models. Considering this fact, RSM and ANN may be applied in combination to improve the optimization of cosmeceutical development and are expected to be used in the coming years as interesting methodologies to achieve the best extraction results as well as increase the quality of developed cosmeceuticals due to their application to optimize their developments.

## Figures and Tables

**Table 1:** Three factor BBD matrix, with three central points.

Run	Factor 1 (X_1_)	Factor 2 (X_2_)	Factor 3 (X_3_)
1	0	0	0
2	0	0	0
3	0	0	0
4	1	0	1
5	−1	0	1
6	1	0	−1
7	−1	0	−1
8	0	1	1
9	0	−1	1
10	0	1	−1
11	0	−1	−1
12	1	1	0
13	−1	1	0
14	1	−1	0
15	−1	−1	0

**Table 2:** Two factor CCD matrix, with one central point.

Run	Factor 1 (X_1_)	Factor 2 (X_2_)
1	0	0
2	0	α
3	0	−α
4	α	0
5	−α	0
6	1	1
7	−1	1
8	1	−1
9	−1	−1

**Table 3:** Three factor CCD matrix, with one central point.

Run	Factor 1 (X_1_)	Factor 2 (X_2_)	Factor 3 (X_3_)
1	0	0	0
2	0	0	α
3	0	0	−α
4	0	α	0
5	0	−α	0
6	α	0	0
7	−α	0	0
8	1	1	1
9	−1	1	1
10	1	−1	1
11	−1	−1	1
12	1	1	−1
13	−1	1	−1
14	1	−1	−1
15	−1	−1	−1

**Table 4:** Application of DoE in advanced extraction methods.

Experimental Design	Technique	Factors	Levels	Runs	Response Variable	Fitting Parameters	Botanical Source	Reference
CCD	MAE	Extraction time (min) Power (W) Solid–liquid ratio (mL/g)	4 to 10 min 10 to 300 W 25 to 100 mL/g	20	Yield TPC TFC	Model adequacy Lack-of-fit *R*^2^	*Hibiscus Sabdariffa*	[62]
		Extraction time (s) Power (W) Ethanol (% *v*/*v*)	30–90 s 350–500 W 20–80%	20	TPC Total anthocyanins	Model adequacy Lack-of-fit *R*^2^ $R_{adj}^{2}$	Peach peels	[68]
		Ethanol concentration (%) Power (W) Extraction time (Min)	40–80% 80–400 W 1–5 min	17	TFC TAA	Model adequacy Lack-of-fit *R*^2^ $R_{adj}^{2}$	Avocado seeds	[69]
	UAE	Solid–liquid ratio Time (min) Power (W)	4–20 1–5 min 100–300 W	20	TPC TFC	Model adequacy Lack-of-fit *R*^2^	Olive leaves	[70]
		Sonication time (min) Ultrasonic intensity (W/cm^2^)	15 to 45 min 0.431 to 0.719 W/cm^2^ 16 to 34 °C	20	TPC	*R* ^2^ $R_{adj}^{2}$	Apple pomace	[71]
		Amplitude (%) Ethanol (%) Temperature (°C) Temperature (°C)	20–50% 15–80% 20–50 °C	16	Yield TAA Hyaluronidase inhibition Bioactive compound content	Model adequacy Lack of fit *R*^2^ $R_{adj}^{2}$	*Opuntia stricta* fruits	[72]
		Solid–liquid ratio (g/mL) Extraction time (min) Temperature (°C)	0.1–0.5 g/mL 1 to 15 min 25 to 80 °C	20	TPC TAA	*R*^2^ $R_{adj}^{2}$ Lack-of-fit	Bitter gourds	[73]
	NPC	Solid–liquid ratio (mL/g) Negative pressure (MPa) Ethanol concentration (%)	30–50 mL/g (−0.035)–(−0.065) MPa 60–80%	20	Genistein extraction	*R* ^2^	*Cajanus cajan* roots	[36]
	PLE	Static time (min) Ethanol concentration (%) Temperature (°C) Acetic acid (%)	0 to 10 min 0 to 100% 40 to 120 °C 0 to 5%	30	Total anthocyanins TFC TPC	*R* ^2^ $R_{adj}^{2}$	*Schius terebinthifolius*	[74]
		Temperature (°C) Extraction time (min)	25–100 °C 10–30 min	11	Yield AChE, BChE LOX TAA	Model adequacy Lack-of-fit *R*^2^ $R_{adj}^{2}$	Orange peels and seeds	[75]
		Ethanol concentration (%) Temperature (°C)	1 to 2% 80 to 160 °C	13	TPCCaffeine retrievalTAA	*R* ^2^	Coffee	[76]
		Ethanol concentration (%) Temperature (°C)	10–90 55–185 °C		TPC Punicalagin content Antimicrobial activity	*R*^2^ $R_{adj}^{2}$ Lack-of-fit	Pomegranate peel	[11]
	SFE	Co-solvent (%) Pressure (bar) Temperature (°C)	5 to 15% 11 to 21 bars 40 to 60 °C	19	Yield TPC TFC DPPH	Lack-of-fit $R_{adj}^{2}$	Mango seed kernels	[77]
		Co-solvent (%) CO_2_ flow (g/min)	10–20% 8–18 g/min	11	TPC Caffeic acid content TAA	*R* ^2^	Potato peels	[78]
		Co-solvent (%) Pressure (MPa) Temperature (°C)	7–11% 15–35 MPa 40–50 °C	16	FRAP DPPH ABTS	Lack-of-fit *R*^2^ $R_{adj}^{2}$	*Castanea sativa* shells	[17]
	EAE	Temperature (°C) Enzyme (AU/g) Reaction time (h) pH	30–50 °C 2–10 AU/g 1–7 h 3–5	28	Yield ABTS TPC	Model adequacy Lack-of-fit *R*^2^ $R_{adj}^{2}$	Bilberry pomace	[79]
		Temperature (°C) Enzyme (FGBU/100 g) Reaction time (min) pH	40 to 60 °C 68 to 268 FGBU/100 g 60 to 18 min 3.5 to 5.5	20	TPC	Lack-of-fit *R*^2^ $R_{adj}^{2}$	*Yerba mate*	[38]
	PEF	Ethanol concentration (%) Extraction time (min) Temperature (°C)	0–100% 30–240 min 20–50°C	15	TPC DPPH	Model adequacy Lack-of-fit *R*^2^	Potato peel	[43]
		Number of voltage Voltage (kV)	40–60 2–6 kV	10	TPC DPPH	Model adequacy Lack-of-fit $R^{2},\;R_{adj}^{2}$ CV	Cinnamon	[44]
BBD	MAE	Ethanol concentration (%) Temperature (°C) Extraction time (min) Solvent volume (mL)	40–80% 40–80 °C 5–40 min 50–80 mL	29	TPC DPPH FRAP Aloin content	Lack-of-fit *R*^2^ $R_{adj}^{2}$	*Aloe vera* skin	[80]
		Ethanol concentration (%)Power (W) Extraction time (s) Solid–liquid ratio	40–80% 100–900 W 30–120 s 1:10–1:70	27	TPC	Model adequacy *R*^2^ $R_{adj}^{2}$	Grapefruit skin	[81]
		Ethanol concentration (%) Power (W) Extraction time (s)Solid–liquid ratio	30–80% 500–900 W 30–120 s 20–40	27	TPC	Model adequacy Lack-of-fit *R*^2^ $R_{adj}^{2}$	Red onions	[82]
	UAE	Solvent/solid ratio (mL/g) Amplitude (%) Time (min) Ethanol concentration (%)	10–30 mL/g 20–40% 20–40 min 40–80%	29	Yield TPC	Lack-of-fit *R*^2^	Meghalayan cherry fruit	[83]
		Temperature(°C)Time (min) Ethanol concentration (%)	70–80 °C 50–70 (min) 50–80%	15	TPC	Lack-of-fit *R*^2^ $R_{adj}^{2}$	Brewers’ spent grain	[84]
		Solvent/solid ratio (% *w*/*v*) Amplitude (W/m^2^) Time (min)	2.5–10% (*w*/*v*) 30–70(W/m^2^) 20–60 min	17	TPC FRAP DPPH ABTS	Model adequacy Lack-of-fit *R*^2^ $R_{adj}^{2}$	Kiwiberry leaves	[4]
		Temperature(°C)Time (min) Ethanol concentration (%)	40–60 °C 20–40 min 25–50%	17	TPC TAA	Lack-of-fit $R_{adj}^{2}$	Argel leaves	[85]
	NPC	Ethanol concentration (%) Solvent/solid ratio (mL/g) Time (min)	20–30% 1:10–1:20 60–80 min	17	Rutin, quercetin, kaempherol, isorhamenitin, narcissin yield	Model adequacy Lack-of-fit *R*^2^	*Flos sophorae immaturus*	[86]
	PLE	Extraction time (min) Solid loading (%) Temperature (°C)	5–25 min 5–15% 150–250 °C	17	TPC TAA	Model adequacy Lack-of-fit *R*^2^	Piper bitle leaves	[87]
		Methanol concentration (%) Temperature (°C) Pressure (bar) pH Purge (s) Flushing (%)	25 to 75% 50 to 100 °C100 to 200 bar 30 to 90 s 3 to 7 50 to 100%	54	TPC Total anthocyanins	*R* ^2^	*Morus nigra*	[88]
		Ethanol concentration (%) Temperature (°C) Pressure (bar)	5–95% 80–160 °C 81–122 bar	15	TPC TFC ABTS	Lack-of-fit *R*^2^ $R_{adj}^{2}$	Mung vean seed coat	[89]
	SFE	Extraction time (min) Pressure (bar) Temperature (°C)	15–45 min 200–300 bar 40–60 °C	15	Yield TPC TAA	Model adequacy *R*^2^ Lack-of-fit	Lavender flowers	[90]
		Co-solvent flow rate (%) Pressure (bar) Temperature (°C)	5–15% 250–350 bar 40–50 °C	15	Yield Carotenoid content	*R*^2^ Lack-of-fit	Mango peel	[91]
		Co-solvent flow rate (%) Pressure (bar) Temperature (°C)	5–15% 100–300 bar 40–60 °C	15	Yield TPC TAA	*R*^2^ $R_{adj}^{2}$ Lack-of-fit	Cacao pod husk	[92]
	EAE	pH Temperature (°C) Extraction time (min)	4 to 6 40 to 60 °C 30 to 90 min	17	TPC Total sugar content	Model adequacy Lack-of-fit *R*^2^	*Ulmus pumila*	[93]
		Enzymolysis time (min) Dosage of cellulose (%) Solvent:sample ratio (mL/g)	75 to 105 min 0.7 to 1.1% 15 to 25 mL/g	17	Polysaccharides retrieval	Model adequacy Lack-of-fit *R*^2^ $R_{adj}^{2}$	Pomegranate	[94]
		Temperature (°C) Enzyme (AU/g) Extraction time (h) pH	25–45 °C 0–200 Au/g 2–6 h 4–5.5	15	TPCABTS	Model adequacy Lack-of-fit *R*^2^ $R_{adj}^{2}$	Grape pomace	[95]
	PEF	Ethanol concentration (%) Pulse number Electric field strength (kV/cm)Solvent:sample ratio (mL/g)	50–70% 8–12 20–30 kV/cm 1:15–1:25	29	Proanthocyanidin recovery DPPH Ferous ion chelating	Model adequacy Lack-of-fit	*Vitis amurensis* seeds	[96]

BBD: Box–Benkhen design; CCD: Central Composite design; EAE: enzyme-assisted extraction; MAE: microwave-assisted extraction; NPC: negative pressure cavitation; PLE: pressurized liquid extraction; PEF: pulsed electric field assisted; *R*^2^: coefficient of determination; $R_{adj}^{2}$: adjusted coefficient of determination; SFE: supercritical fluid extraction; UAE: ultrasound-assisted extraction; TPC: total phenolic content; TFC: total flavonoid content; FRAP: ferric reducing antioxidant power; TAA: total antioxidant activity; AChE: acetylcholinesterase inhibition; BChE: buthylcholinesterase; LOX: lipoxygenase inhibition.

**Table 5:** Application of experimental design for optimization of cosmeceutical formulations.

Formulation	Activity	Extract	Factors	Response Variables	Optimal Conditions	Ref.
Safflower oil-based nanoemulsions	Anti-acne	Mango kernel	HLB value PEG-7 glyceryl cocoate Surfactant/oil ratio	Droplet size PDI Z-potential	HLB: 10 PEG-7 glyceryl cocoate: 2% Surfactant/oil ratio: 1.9:1	[112]
Castor oil-kojic monooleate nanoemulsion	Skin hyperpigmentation	Kojic monooleate acid	Time of shear Speed of shear Sonication time	Particle size	Time of shear: 11.16 min Speed of shear: 218 rpm Sonication time: 16.75 min	[114]
Rapeseed oil-mineral oil-isopropyl palmitate emulsion	Photoprotection	Grape pomace	Concentration of sunscreens filtersConcentration of extract	SPF AA UVA transmittance UVB transmittance UVA/UVB ratio	Concentration of sunscreens filters: 11.5% Concentration of extract: 10%	[115]
Wheat germ oil emulsion	-	Wheat sprout	Emulsifier Emulsification time Extract concentration	Droplets size Viscosity Emulsion stability index	Emulsifier: 7.7% Emulsification time: 23.6 min Extract concentration: 3.9%	[120]
Virgin coconut oil emulsion	-	*Chromoleana odorata*	Coconut oil/water ratio Emulsifier concentration Speed	pH Droplet size	Coconut oil/water ratio: 5:95 Emulsifier concentration: 3% Speed: 7500 rpm	[116]
Vitamin E-rich red palm oil-based nanoemulsion	-	-	Tween 80/Span 80 concentration Glycerol Homogenization pressure	Droplet size PDI	Tween 80/Span 80 concentration: 63/37 Glycerol: 20% Homogenization pressure: 500 bar	[118]
Coconut oil emulsion	-	*Flos Sophorae Immaturus*	Emulsification time Speed Amounts of the emulsifier Amounts of additive	Droplets size Viscosity Emulsion stability index	Emulsification time: 17.8 min Speed: 5505 rpm Amounts of the emulsifier: 2.28% Amounts of additive: 1.05%	[121]
